# Evaluation of community provision of a preventive cardiovascular programme - the National Health Service Health Check in reaching the under-served groups by primary care in England: cross sectional observational study

**DOI:** 10.1186/s12913-017-2346-5

**Published:** 2017-06-14

**Authors:** Maria Woringer, Elizabeth Cecil, Hillary Watt, Kiara Chang, Fozia Hamid, Kamlesh Khunti, Elizabeth Dubois, Julie Evason, Azeem Majeed, Michael Soljak

**Affiliations:** 1 Department of Primary Care and Public Health, Imperial College London, Charing Cross Campus, The Reynolds Building, St Dunstan’s Road, London, W6 8RP UK; 2 Diabetes Research Centre, Leicester Diabetes Centre, University of Leicester, Leicester, LE5 4PW UK; 3 Health Diagnostics Ltd., Suite C, The Quadrant,, Sealand Road,, Chester, CH1 4QR UK

**Keywords:** Cardiovascular disease, Primary prevention, Health inequalities, Young, Deprived, Ethnic minorities

## Abstract

**Background:**

Cardiovascular disease (CVD) is the leading cause of premature mortality and a major contributor of health inequalities in England. Compared to more affluent and white counterparts, deprived people and ethnic minorities tend to die younger due to preventable CVD associated with lifestyle. In addition, deprived, ethnic minorities and younger people are less likely to be served by CVD prevention services. This study assessed the effectiveness of community-based outreach providers in delivering England’s National Health Services (NHS) Health Check programme, a CVD preventive programme to under-served groups.

**Methods:**

Between January 2008 and October 2013, community outreach providers delivered a preventive CVD programme to 50,573 individuals, in their local communities, in a single consultation without prescheduled appointments. Community outreach providers operated on evenings and weekends as well as during regular business hours in venues accessible to the general public. After exclusion criteria, we analysed and compared socio-demographic data of 43,177 Health Check attendees with the general population across 38 local authorities (LAs). We assessed variation between local authorities in terms of age, sex, deprivation and ethnicity structures using two sample t-tests and within local authority variation in terms of ethnicity and deprivation using Chi squared tests and two sample t-tests respectively.

**Results:**

Using Index of Multiple Deprivation, the mean deprivation score of the population reached by community outreach providers was 6.01 higher (*p* < 0.05) than the general population. Screened populations in 29 of 38 LAs were significantly more deprived (*p* < 0.05). No statistically significant difference among ethnic minority groups was observed between LAs. Nonetheless some LAs – namely Leicester, Thurrock, Sutton, South Tyneside, Portsmouth and Gateshead were very successful in recruiting ethnic minority groups. The mean proportion of men screened was 11.39% lower (*p* < 0.001) and mean proportion of 40–49 and 50–59 year olds was 9.98% and 3.58% higher (*p* < 0.0001 and *p* < 0.01 respectively) than the general population across 38 LAs.

**Conclusions:**

Community-based outreach providers effectively reach under-served groups by delivering preventive CVD services to younger, more deprived populations, and a representative proportion of ethnic minority groups. If the programme is successful in motivating the under-served groups to improve lifestyle, it may reduce health inequalities therein.

## Background

Despite substantial reductions in mortality, CVD remains the leading cause of premature death in England and a major contributor of health inequalities perpetuated by socioeconomic status, ethnicity and geographical location. While the government’s expenditure on health as percent of GDP has risen from 6.8% in 1995 to 9.6% in 2010, health inequalities remain [1, 2]. In England, worse outcomes from CVD have been reported in more deprived areas with 50% higher mortality in the most deprived fifth of the population compared with the least deprived [2], among South-Asian and Black ethnic groups who experience the highest mortality rates from coronary heart disease and stroke respectively [3, 4] and in the North of England compared with the South [5]. The European Guidelines on CVD prevention cite an increase in CVD risk and mortality among socially deprived and ethnic minorities across Europe [6]. Relative to other European countries, between 1990 and 2010 years of life lost due to premature mortality in England have worsened for men and women younger than 55 years in whom rates of decrease in heart disease mortality have slowed [1, 7]. Much of the CVD burden is associated with lifestyle factors is largely preventable [3]. Preventing the premature onset of CVD while reducing health inequalities is an important objective for England’s National Health Service (NHS).

The NHS Health Check programme, introduced by the Department of Health in April 2009, is a national CVD risk assessment and management scheme aimed at preventing heart disease, stroke, diabetes and kidney disease, whilst reducing health inequalities among all CVD free individuals 40–74 years living in England. The programme, offered primarily in general practice, aims to tackle the premature burden of the disease by educating people about adopting a healthy lifestyle while referring those at increased risk for further services and prescribing lipid lowering medication. Patients found to have pre-existing disease are referred for formal diagnosis and enter established care pathways [8, 9]. It was estimated that the programme could prevent 1600 heart attacks and strokes, at least 650 premature deaths and over 4000 new cases of diabetes per year. In addition 20,000 new cases of diabetes or kidney disease could be detected earlier. The estimated cost per quality adjusted life year was £3000. The NHS Health Check programme at full implementation ranging from £180 to £243 million a year is less than 1% of the cost of CVD to the NHS and the UK economy estimated at £30 billion per year [10, 11]. In light of an aging population and increasingly constrained public expenditures, NHS needs to move away from treatment to earlier detection and management of CVD. Critics of the Health Check programme cite a lack of evidence for population based health checks and the potential to exacerbate health inequalities [12, 13].

Groups under-served in primary CVD prevention programmes in primary care include deprived people, ethnic minorities, and younger people [14–17]. Reaching out to deprived populations and ethnic minorities who are at increased risk of CVD is important for reversing health inequalities. Making lifestyle changes in middle age has the potential to increase life expectancy overall [18]. Between 2008 and 2013, public health teams in primary care trusts (PCTs) and subsequently local authorities (LAs) commissioned community outreach providers to conduct Health Checks in local communities with the aim of reducing health inequalities, by targeting more deprived individuals and people from ethnic minority groups. In 2013, Public Health England asserted their commitment to improving programme uptake overall, with improved uptake particularly in deprived communities and among ethnic minority groups [19].

As the Health Check programme comes under increased scrutiny at the time of financial austerity, more evidence is needed to show what works well in delivering the programme. In light of national evaluations of Health Check provision in general practice showing very modest to non-significant differences in Health Check attendance by level of deprivation and mixed results in terms of targeting ethnic minorities across England [20, 21], a different approach may be needed to tackle health inequalities in relation to CVD. Local studies suggest that community-led primary prevention of CVD may be an effective response to tackle low uptake by under-served groups [22–26]. The aim of this study was to evaluate the potential of the Health Check programme when offered in local communities to reduce health inequalities by targeting under-served groups in primary care. For this purpose Health Check attendees across 38 (of 326 LAs) were analysed. The resident population of these 38 LAs was 12.81% of the total English population in 2011 [27].

## Methods

### Study design

A cross-sectional observational study design was used to conduct the study. The study population consisted of people who received a community provided NHS Health Check specifically using Health Options® software and point of care testing between January 2008 and October 2013. Data on 50,573 Health Check attendees was obtained under a license agreement from Health Diagnostics. The aim of this study was to assess whether the Health Check attendees recruited by community providers differed from the general population in gender, age, ethnicity and socio-economic status.

### Setting and mode of health check delivery

Providers of opportunistic Health Checks operated in the local communities on evenings and weekends in addition to regular business hours. Health Checks were delivered in pharmacies, community centres, places of worship, businesses, council offices, libraries, shopping centres, village halls, schools and football stadiums. Pharmacy staff delivered 59.90% of all community outreach Health Check consultations, private companies 25.30%, health improvement foundation trust 10.58%, and LA occupational health departments 4.22%. Providers of Health Checks included pharmacists, pharmacy technicians and pharmacy counter staff, nurses, health improvement staff, fitness instructors, health promotion specialists, and occupational health nurses. All providers using Health Options® software and point of care testing, were trained to follow standardised procedures to collect routine Health Check data.

Unlike standard clinical systems such as EMIS, INPS-Vision and TPP-SystmOne used in general practice primarily for routine collection of all clinical data, Health Options® software is specifically used for delivering the NHS Health Check Programme by community outreach providers. This patient centred software was used to communicate CVD risk to Health Check attendees with graphics and “what if” scenarios illustrating the impact of lifestyle change on an individual’s 10 year predicted risk of CVD. Whereas Health Checks provided at the general practice could involve several visits if blood samples are sent for laboratory testing, community outreach providers completed Health Checks in a single visit using point of care testing.

### Study participants

We analysed anonymised data from 50,573 individuals who received a Health Check carried out by community outreach providers in 90 LAs from 30th January 2008 to 31st October 2013. The following exclusion criteria were applied: 581 individuals younger than 40 years of age were excluded as these individuals would not be eligible for the NHS Health Check programme, 6536 individuals who could not be matched to a LA, and 279 individuals that came from LAs constituting fewer than 0.10% of 2011 general population aged 40–74 as these LAs contained too few individuals for inclusion for comparative analyses. A minimum benchmark for inclusion as proportion of the general population data was chosen as some LAs contained larger populations than others. A cut off was chosen so as to have a minimum standard for inclusion while maximizing the number of LAs included into the analysis. The majority of LAs with less than 0.10% of the general population contained data in single digits which would not be suitable for inter-LA comparisons. Following the exclusion criteria, data on 43,177 individuals from 38 LAs across eight regions of England were analysed.

### Assessment of gender, age and ethnicity

The total population, according to gender and aged 40–74 years old in the 38 LAs (*n* = 2,793,398), was obtained using the Office for National Statistics (ONS) 2011 Census population estimates by single year of age and sex for each LA in England [28, 29]. Ethnicity data by age in the 38 LAs was obtained using ONS 2011 ethnic group by sex by age [30]. Ethnicity groupings were built upon the 2001 Census categories as suggested in the Health Check secondary use dataset [31]. Ethnicity groupings included White (composed of British or Mixed British, Irish and Any Other White Background), Mixed (White and Black Caribbean, White and Black African, White and Asian, and Other Mixed Background), Asian (composed of Indian, Pakistani, Bangladeshi or Other Asian Background), Black (composed of Caribbean, African and Other Black Background), Other Ethnic Groups (Chinese, Other), Ethnic Category Not Stated and Ethnic Category Unknown. The ethnic breakdown of the general population was restricted to 40–74 year olds in the 38 LAs (*n* = 2,793,077). Gender, age and ethnicity were self-reported by Health Check attendees. Chi squared tests were used to compare ethnicity among Health Checks attendees with the general population in each LA. Two sample t-tests were used to compare the age, sex and ethnicity structures between LAs among Health Check attendees and the general population.

### Assessment of deprivation

To assess levels of deprivation in the general population, we used an average Index of Multiple Deprivation (IMD) 2010 scores by patient post code for the resident population in each LA as reported by the Department for Communities and Local Government [32]. IMD measures relative levels of deprivation in small areas of England using 38 separate indicators organised across seven distinct domains of deprivation (income, employment, health and disability, education skills and training, barriers to housing and services, living environment and crime) [33]. IMD scores and quintiles are defined at the Lower Layer Super Output areas in England [34]. As only 44.97% of Health Check attendees had a recorded postcode of residence while 86.90% had a postcode of general practice, to assess levels of deprivation among Health Check attendees, we used general practice associated IMD scores and quintiles. Using these IMD scores, we calculated population weighted average IMD 2010 scores in each LA. We compared average IMD 2010 scores within each LA between Health Check attendees and the general population as well as across all 38 LAs using two sample t tests. Data were analysed using Stata/SE Version 12.

## Results

### Baseline characteristics

**Fig. 1:**
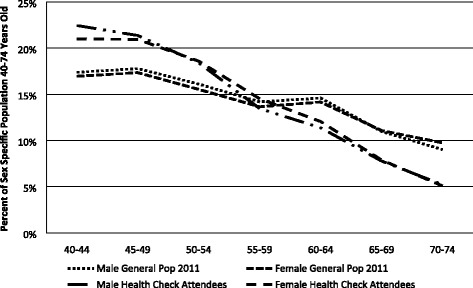
Age by sex as percent of sex specific population

Among 43,177 Health Check attendees from 38 LAs, the proportion of men was 38.15% (vs. 49.12% in the general population), the proportion of 40–49 year olds was 42.58% (vs. 35.03% in the general population), among 50–59 it was 32.58% (vs. 29.98% in the general population), and among 60–74 it was 24.85% (vs. 34.99% in the general population). Compared to the general population data, Health Check attendees were younger than the general population irrespective of sex. (Fig. 1)

Among Health Check attendees, 88.24% were White (vs 92.71% in the general population), 0.45% were Mixed (vs 0.74% in the general population), 7.60% were Asian or Asian British (vs 4.25% in the general population), 1.39% Black or Black British (vs 1.39% in the general population), 0.70% Other Ethnic Group (vs 0.91% in the general population), 0.26% Ethnic Category Unstated and 1.35% Ethnic Category Unknown.

Community providers served larger proportions of the population in North of England (North East, North West and Yorkshire and the Humber): 66.52% than in S Midlands and East of England (West Midlands, East Midlands and East of England): 21.20%, or South of England (London and South East): 12.24%. Nearly half of all examined LAs were in the most deprived fifth using ranks of average IMD 2010 scores (ranks of 1 to 326 are from most to least deprived) [35]. A large segment of the study population was registered in general practices with the highest levels of deprivation. From most to least deprived using IMD quintiles, the proportion of people in each fifth was 45.03%, 21.49%, 15.38%, 12.83%, and 5.28%. Among Health Check attendees, in the most deprived fifth, 40–49 year olds made up 44.79% of the population, followed by 32.88% of 50–59 year olds and 22.32% of the 60–74 year olds. The proportion of men to women did not vary by deprivation.

### Main findings

We compared demographics between LAs among Health Check attendees and general population using two-sample t-tests. The mean proportion of men was lower among the Health Check attendees (37.79%) compared to the general population (49.18%) (*p* < 0.001, Table 1). The mean proportion of 40–49 and 50–59 year olds was higher among Health Check attendees (43.63% and 33.35%) than among general population (34.65% and 29.77%, *p* < 0.001 and *p* < 0.01 respectively). Conversely, the proportion of 60–74 year olds was larger (*p* < 0.001) among general population (35.58%) compared to Health Check attendees (22.01%).

**Table 1:** Inter local authority comparison of Health Check attendees with the general population

Variable		Health Check Attendees Mean (95%CI)	*p*-value	General Population Mean (95% CI)
Deprivation	IMD	30.15 (25.97, 34.33)	*	24.14 (21.22–27.06)
Ethnicity	White	92.15% (87.90%, 96.39%)		94.03% (91.08%, 96.98%)
	Mixed	0.50% (0.31%, 0.69%)		0.65% (0.48%, 0.83%)
	Asian	4.15% (0.70%, 7.60%)		3.38% (1.15%, 5.61%)
	Black	1.62% (0.47%, 2.77%)		1.17% (0.58%, 1.76%)
	Other	0.56% (0.32%, 0.80%)		0.77% (0.53%, 1.01%)
Age	40–49	44.63% (41.65%, 47.62%)	***	34.65% (33.73%, 35.57%)
	50–59	33.35% (31.62%, 35.09%)	***	29.77% (29.39%, 30.15%)
	60–74	22.01% (18.56%, 25.46%)	***	35.58% (34.50%, 36.65%)
Sex	Male	37.79% (36.17%, 39.42%)	***	49.18% (48.95%, 49.42%)
	Female	62.21% (60.58%, 63.83%)	***	50.82% (50.58%, 51.05%)

*Statistically significantly different at *p* < 0.05; **statistically significantly different at *p* < 0.01; ***statistically significantly different at *p* < 0.001. CI – confidence interval

Between 38 LAs, there was no statistically significant difference in the mean proportion of ethnic minority groups among Health Check attendees and the general population. The mean proportion of those with Asian ethnicity was 4.15% among Health Check attendees compared to 3.38% in the general population. The mean proportion with Black ethnicity was 1.62% among Health Check attendees compared to 1.17% in general population. Nonetheless there was substantial inter-LA variability. (Table 2) The Asian population within LAs varied from 0.00% to 61.73% among Health Check attendees and between 0.27% and 36.77% in the general population. The proportion with Black ethnicity between LAs varied from 0.00% to 17.73% among Health Check attendees and between 0.04% and 6.97% in the general population.

**Table 2:** Inter local authority summary statistics

Health Check Attendees								
Variable		Mean	SD	Min	Max	25th %ile	50th %ile	75th %ile
Deprivation	IMD	30.15	12.71	8.03	55.67	19.36	28.69	36.32
Ethnicity	White	92.15%	12.91%	33.49%	100.00%	91.91%	97.64%	98.84%
	Mixed	0.50%	0.59%	0.00%	2.26%	0.00%	0.29%	0.69%
	Asian	4.15%	10.50%	0.00%	61.73%	0.00%	1.02%	3.75%
	Black	1.62%	3.49%	0.00%	17.73%	0.00%	0.34%	0.89%
	Other	0.56%	0.73%	0.00%	3.19%	0.00%	0.31%	0.79%
Age	40–49	44.63%	9.09%	24.47%	61.76%	38.14%	43.33%	52.50%
	50–59	33.35%	5.28%	22.92%	48.44%	29.52%	33.25%	36.22%
	60–74	22.01%	10.50%	3.13%	43.92%	12.47%	22.75%	27.78%
Sex	Male	37.79%	4.94%	27.01%	46.94%	34.62%	37.18%	40.34%
	Female	62.21%	4.94%	53.06%	72.99%	59.66%	62.82%	65.38%
General Population								
Variable		Mean	SD	Min	Max	25th %ile	50th %ile	75th %ile
Deprivation	IMD	24.14	8.87	9.62	43.45	19.44	23.85	29.48
Ethnicity	White	94.03%	8.98%	55.15%	99.31%	94.19%	97.64%	98.82%
	Mixed	0.65%	0.52%	0.21%	2.67%	0.31%	0.48%	0.68%
	Asian	3.38%	6.79%	0.27%	36.77%	0.43%	1.05%	2.50%
	Black	1.17%	1.80%	0.04%	6.97%	0.10%	0.26%	1.14%
	Other	0.77%	0.72%	0.17%	3.03%	0.25%	0.48%	0.96%
Age	40–49	34.65%	2.80%	29.58%	40.31%	32.67%	34.36%	36.46%
	50–59	29.77%	1.14%	27.04%	32.36%	29.01%	29.55%	30.47%
	60–74	35.58%	3.28%	30.04%	41.89%	33.24%	35.21%	37.70%
Sex	Male	49.18%	0.72%	47.39%	50.62%	48.77%	49.22%	49.61%
	Female	50.82%	0.72%	49.38%	52.61%	50.39%	50.78%	51.23%

Note : *SD* = standard deviation, *Min* = minimum, *Max* = maximum, %ile – percentile

Some LAs were very successful in recruiting ethnic minority groups, whilst others were less successful. In Leicester 61.73% of Health Check attendees were of Asian ethnicity (vs. 36.77% of the general population). In Thurrock, 4.61% of all Health Check attendees were Asian and 17.73% were Black (vs. 2.32% and 5.42% of the general population). In Sutton, 9.31% of Health Check attendees were Black (vs. 4.38% of the general population). In South Tyneside, 3.75% of all Health Check attendees were Asian and 0.36% were Black (vs. 1.09% and 0.17% of the general population). In Portsmouth 3.87% of Health Check attendees were Asian and 3.31% were Black (vs. 2.50% and 0.83% of the general population). In Gateshead 2.19% of Health Check attendees were Asian and 0.39% were Black (vs. 0.80% and 0.24% of the general population). Using a Chi Squared test, these differences were statistically significant (*p* < 0.0001).

*SD* standard deviation, *Min* minimum, *Max* maximum, *%ile* percentile

Compared to the general population, mean IMD 2010 score across the 38 LAs was 6.01 higher among Health Check attendees at *p* < 0.05. Comparing each LA’s Health Check population mean with the general population mean using two sample t-tests, 29 of the 38 LAs contained populations that were significantly more deprived than the general population at *p* < 0.05 (Table 3). These results combined with the wider range of average IMD 2010 scores among Health Check attendees between 38 LAs suggest that providers targeted more deprived communities in the LAs that they served.

**Table 3:** Intra LA comparison of deprivation of Health Check attendees with the general population

	Health Check Attendees Mean IMD (95% CI)	*p*-value	General Population Mean IMD	General Population Rank of Average Score
East Midlands				
Leicester	34.17 (33.73, 34.62)	*	33.65	25
Nottingham	47.36 (46.60, 48.11)	***	34.42	20
East of England				
Basildon	24.17 (23.58, 24.76)	***	20.56	131
Brentwood	8.03 (7.59, 8.47)	***	9.62	294
Thurrock	25.51 (23.70, 27.31)	***	19.45	143
London				
Hillingdon	18.83 (18.51, 19.14)	***	19.81	138
Richmond upon Thames	10.21 (9.99, 10.43)		10.12	285
Sutton	19.31 (18.68, 19.93)	***	15.43	196
North East				
County Durham	24.20 (23.82, 24.59)	***	26.41	62
Darlington	27.76 (25.23, 30.29)		25.41	75
Gateshead	33.16 (32.59, 33.73)	***	29.48	43
Hartlepool	55.67 (54.21, 57.14)	***	33.68	24
Middlesbrough	51.16 (49.49, 52.82)	***	37.62	8
Newcastle upon Tyne	31.84 (27.88, 35.81)		29.74	40
North Tyneside	24.89 (21.98, 27.80)		22.24	113
Redcar and Cleveland	36.23 (35.21, 37.26)	***	28.55	48
South Tyneside	37.35 (36.92, 37.79)	***	28.35	52
Stockton-on-Tees	43.47 (42.14, 44.81)	***	23.46	100
Sunderland	36.32 (35.83, 36.82)	***	29.46	44
North West				
Allerdale	19.36 (18.49, 20.24)	***	22.30	111
Barrow-in-Furness	42.00 (37.88, 46.11)	***	30.92	32
Carlisle	28.28 (27.42, 29.14)	***	22.56	109
Copeland	34.14 (32.54, 35.75)	***	22.56	78
Eden	20.71 (20.05, 21.37)	***	14.07	211
Knowsley	54.52 (54.18, 54.86)	***	41.01	5
Liverpool	51.56 (50.52, 52.61)	***	43.45	1
Sefton	29.10 (28.42, 29.78)	***	24.25	92
South Lakeland	13.51 (13.26, 13.76)	***	12.42	242
South East				
Portsmouth	50.17 (48.66, 51.68)	***	25.41	76
West Midlands				
Bromsgrove	16.29 (15.12, 17.45)	***	10.38	281
Coventry	29.86 (29.04, 30.68)	***	28.44	50
Malvern Hills	14.57 (14.23, 14.92)	***	13.49	223
Redditch	35.77 (30.85, 40.69)	***	21.85	117
Worcester	24.51 (23.09, 25.93)	***	19.44	144
Wychavon	16.94 (15.41, 18.46)	***	13.19	229
Wyre Forest	24.51 (22.74, 26.28)	***	21.04	124
Yorkshire and the Humber				
Hambleton	16.66 (15.06, 18.27)	***	10.97	264
Kingston upon Hull, City of	33.59 (32.74, 34.43)	***	37.53	10

*Statistically significantly different at *p* < 0.05; ** statistically significantly different at *p* < 0.01; *** statistically significantly different at *p* < 0.001

## Discussion

### Main findings

This is the first study looking at community outreach provision of Health Checks across multiple LAs in England with respect to general population statistics. Community providers of Health Checks worked in some of the most deprived LAs across England and targeted more deprived people. Individuals in the most deprived fifth made up nearly half of all Health Check attendees. Among those, nearly half of the population were under 50 years old. Offering the Health Check Programme on evenings and weekends may have enabled a substantial proportion of younger employed people to take part in the Health Check. More women than men were served by community providers. Community providers targeted a nationally representative proportion of ethnic minorities across the LAs that they served, with some LAs, notably Leicester, Thurrock, Sutton, South Tyneside, Portsmouth and Gateshead being particularly successful at targeting ethnic minorities.

### What is already known on this topic

As a result of NHS restructuring and major organizational changes from PCTs to LAs taking the responsibility for NHS Health Checks, the NHS Health Check programme was not uniformly implemented across England. In 2013, 27 of 151 PCTs offered NHS Health Checks to fewer than 10% of eligible individuals [21]. Although offer and uptake figures are reported across England at the LA level, these figures are not broken down by mode of provision (general practice or community outreach) nor by demographics (age, ethnicity and deprivation) [36]. There is no data and no previous studies on community outreach provision of NHS Health Checks across England, estimated to be less than 10% of all NHS Health Checks [21]. Although general practice data exists across England, it can only be examined at the regional level [20]. Doing so important differences between LAs in implementing the programme are lost to analysis and the programme may appear to be ineffective as a whole.

The results are mixed with respect to reporting the programme’s potential in reducing health inequalities when offered in general practice. Two nationally representative retrospective studies reported lower than expected coverage for the program as a whole (defined as Health Check attendance of the eligible population) and higher coverage among older populations. [20, 21] Whereas one of these studies found coverage of the programme to be equitable by deprivation with lower coverage among ethnic minorities [20], another study suggested slightly higher programme coverage among ethnic minorities and among socially deprived populations [21]. A study of 3 PCTs across East London showed increasing coverage in the first 3 years with equitable coverage across all deprivation groups and ethnic minorities [37]. Local studies suggest low uptake of the programme (defined as Health Check attendance among the eligible population invited for a Health Check) when offered in general practice. Higher uptake was reported by wealthier individuals, Black and Asian groups, and older people [14, 15, 38, 39].

Barriers to accessing preventive CVD services in general practice within England include the inconvenience of attending pre-scheduled appointments during regular working hours for working age people [37, 38], dissatisfaction among minorities and language barriers among Asians, [25, 40] no uniform availability of point of care testing allowing the Health Check to be completed in a single consultation [41], lack of patient centred software designed to be viewed by patients in consultation with clinicians [42–45] and a shortage of general practices in some of the most deprived areas [46, 47]. Reducing barriers to accessing preventive services is a major challenge for primary care [48]. Despite the UK government’s initiative to increase the availability of general practices in deprived areas, general practice coverage in areas associated with increased levels of deprivation was well below the national average in 2008 [46]. Hence uniform programme coverage by deprivation observed in general practice may not mean equitable coverage particularly in the most deprived areas of England. General practice delivery of the Health Check programme may exacerbate health inequalities by benefiting those in higher socioeconomic groups while under serving younger people and ethnic minorities. [49, 40, 48, 50, 51] Reaching the hard to reach groups may require a more proactive community based approach [52].

Evidence supporting the feasibility and acceptability of community outreach screening is increasing [53, 54]. Increasing accessibility of drop in preventive services at community venues at times outside standard working hours was suggested to make access easier for under-served groups [22, 55]. The use of an out of hours vascular screening service in pharmacies in Birmingham was successful in capturing more working age men [23]. Successful recruitment of Asian population was reported in a local evaluation of community pharmacy provision of vascular risk assessment in Leicester [25]. Effective targeting of younger individuals and more deprived areas and communities was reported with lay health trainer led community provision of Health Checks in Durham [24].

### What this study adds

This is the first study describing the effectiveness of community provision of Health Checks across multiple LAs in England to target under-served groups in primary care. Community provision of care may play a key role in reducing the barriers to preventive services among the under-served groups. Unlike general practice Health Checks, community Health Checks were offered at more convenient times, in more convenient locations, in local languages, while minimizing the loss to follow up with the use of mobile technology and point of care testing. Offering the Health Check programme in a range of community venues outside of conventional general practice hours enabled many working age people who may not regularly access general practice services to receive a Health Check. Pharmacy staff fluent in a number of Asian languages may have been particularly effective in recruiting higher proportions of Asian men and women in Leicester. Community based Health Checks completed in a single visit using point of care testing, minimized the loss to follow up that may occur when attendees are required to return for blood tests and clinical results. Using patient centred Health Options® software to present CVD risk with graphics and risk lowering scenarios, community providers of Health Checks engaged with a large proportion of socioeconomically deprived individuals.

In light of persisting ethnic and socioeconomic health inequalities and a slowing rate of CVD mortality decline among under 55 year olds in England [1, 2, 56–58], a new approach is needed to tackle health inequalities in England. Community provision of preventive services is a viable alternative to deliver Health Checks in general practice in order to better serve the needs of the most marginalized members of society. If successful in persuading people to improve their lifestyle, community provision of the programme may reduce health inequalities associated with CVD.

### Limitations

The main limitation of this study is that it is not nationally representative as the sample of Health Check attendees was not drawn from English population at random. Taking a random sample of data would require a much larger initial sample size. The lack of comparison of uptake and coverage with general practice data proved to be another limitation. As invitations to participate in community Health Checks are not routinely recorded data, it was not possible to estimate uptake. In addition, eligible population across England was not reported until 2011 making it difficult to report coverage from the outset of the programme in 2009. Another possible limitation of this analysis is the comparison of deprivation among Health Check attendees using practice postcode with the postcode of residence in the general population data. Perhaps some of the heterogeneity of deprivation data was lost in using general practice post code among Health Check attendees. Nonetheless it is accepted practice in the absence of postcode of residence data to report deprivation among programme attendees at the practice level [49].

## Conclusions

Targeted community implementation of the NHS Health Check programme by outreach providers could contribute to reducing health inequalities associated with CVD. The results of this study suggest that using community outreach providers is an effective approach to reach younger people, more deprived areas and individuals while recruiting a representative proportion of ethnic minority groups across England. Expansion of community outreach service is recommended to reach out to the most marginalized populations. Further research is needed to see whether the NHS Health Check programme is successful in preventing CVD among attendees.

## Abbreviations

CI: Confidence interval
CVD: Cardiovascular disease
IMD: Index of multiple deprivation
LA: Local authority
NHS: National Health Service
